# Prevalence of cardiac myosin-binding protein C3 mutations in Maine Coon cats with hypertrophic cardiomyopathy

**DOI:** 10.14202/vetworld.2022.502-508

**Published:** 2022-02-27

**Authors:** Pratch Sukumolanan, Soontaree Petchdee

**Affiliations:** 1 Veterinary Clinical Studies Program, Graduate School, Kasetsart University, Kamphaeng Saen Campus, Nakorn Pathom, 73140, Thailand; 2 Department of Large Animal and Wildlife Clinical Sciences, Faculty of Veterinary Medicine, Kasetsart University, Kamphaeng Saen Campus, Nakorn Pathom, 73140, Thailand

**Keywords:** feline, hypertrophic cardiomyopathy, mutation, myosin-binding protein

## Abstract

**Background and Aim:**

Hypertrophic cardiomyopathy (HCM) is a common heart problem that affects many cats. Although cats with HCM are symptomatic, some die suddenly or develop congestive heart failure. Therefore, this study aimed to estimate the prevalence of myosin-binding protein C3 (*MYBPC3*), A31P, and A74T polymorphisms in Maine Coon cats to assess risk factors for diagnosing HCM in cats.

**Materials and Methods:**

Forty-nine Maine Coon cats of at least 10 months of age were enrolled in this study. First, clinical parameters, such as heart rate, systolic blood pressure, and echocardiography, were evaluated. Then, polymerase chain reaction, followed by DNA sequencing, was conducted using specific primers for amino acid substitutions caused by genetic variants of *MYBPC3*-A31P and -A74T polymorphisms.

**Results:**

Investigations showed that the prevalence of *MYBPC3*-A31P and -A74T mutations in this study was 16.33% and 24.45%, respectively. Moreover, HCM in cats with *MYBPC3*-A31P and A74T mutations increased with age, body weight, high heart rate, and prolonged isovolumic relaxation time.

**Conclusion:**

Therefore, we propose that Maine Coon cats develop HCM due to multiple genetic factors and underlying clinical characteristics in individual cats. Furthermore, relaxation time assessments can be a sensitive technique for HCM screening during its preclinical phase and can help identify the risk of developing HCM. However, further studies are warranted to evaluate the effect of *MYBPC3* mutations on the phenotypic expression of HCM.

## Introduction

Hypertrophic cardiomyopathy (HCM) is an inherited heart problem in the feline population. It is commonly caused by autosomal dominant gene mutations that encode various cardiac sarcomere proteins. Mutations in cardiac myosin-binding protein C3 (*MYBPC3*), such as A31P and A74T gene polymorphisms, have been proposed to cause HCM in Maine Coon cats [1-3]. Moreover, while the worldwide prevalence of *MYBPC3*-A31P is approximately 34-42%, the *MYBPC3*-A74T mutation is 35% in Maine Coon and 62% in other breeds [3-5]. A cohort study revealed that 100% of cats with homozygous *MYBPC3*-A31P mutations developed HCM within 5 years of age [6]. Therefore, the American College of Veterinary Internal Medicine (ACVIM) consensus statement guidelines for classifying, diagnosing, and managing cardiomyopathies in cats recently recommended evaluating sarcomeric mutations of *MYBPC3* before breeding to decrease this mutation in the feline population [7,8].

The previous studies have also suggested that the prevalence of HCM was higher in older cats with higher body weights [9,10]. Therefore, as a screening method, the feline NT-proBNP plasma concentration can be used to screen for dogs and cats with cardiomyopathy. Furthermore, the atrial natriuretic peptide is proposed to help diagnose HCM in Maine Coon cats as an early screening tool for HCM [11-15]. However, echocardiography is the primary diagnostic tool for HCM as recommended in the ACVIM guidelines, characterized by increased left ventricular wall thickness. In addition, a recent study demonstrated diastolic myocardial motion using tissue Doppler imaging as an early marker of HCM [16-18]. However, only a few reports on the risk factors and tools for the early detection of HCM in cats exist [19-21].

Therefore, this study aimed to estimate the prevalence of HCM in Maine Coon cats and evaluated risk factors of HCM onset in cats.

## Materials and Methods

### Ethical approval and Informed consent

The study was approved by the Ethical Committee for Animal Experiments, Kasetsart University, Thailand (ACKU 62-VET-059). This study involved the use of non-experimental animals only. Verbal consent was obtained from all the owners and no animals or humans are identifiable within this publication,

### Study period and location

This study was conducted from April 2019 to May 2020 at the Kasetsart University Teaching Hospital Kamphaeng Saen, Faculty of Veterinary Science, Kasetsart University.

### Animal

Forty-nine client-owned Maine Coon cats were enrolled in this study. As the inclusion criteria, only cats at least 10 months of age were recruited in this study. However, cats were excluded if they had the following conditions: (1) Secondary left ventricular hypertrophy, such as cardiac structural abnormalities (aortic stenosis), (2) hyperthyroidism (total T4 level >3.8 μg/dL), (3) systemic hypertension (systolic blood pressure >160 mmHg), (4) pregnancy or were lactating, and (5) previous or current chronic diseases, such as chronic kidney disease, diabetes mellitus, or status epilepticus.

### Clinical examination

Cats were subjected to a complete physical examination to evaluate their general conditions. Hence, 49 Maine Coon cats were enrolled, after which their clinical data, including age, sex, and body weight, were collected. Subsequently, clinical parameters, such as heart rate and blood pressure, were also evaluated. Then, indirect blood pressure measurements using a Doppler device (Parks Medical Electronics, USA) were performed in triplicate, followed by a complete blood count and serum biochemistry profile for each cat as an essential routine health check-up.

### DNA sequencing

With minimal restraints, blood samples (2-3 mL) were collected from the venous vessel of each cat. Then, these samples were stored at −20°C until DNA extraction. Subsequently, nucleic acid was extracted from blood samples (200 μL) using a DNA extraction kit (Blood Genomic DNA Extraction Mini Kit, Favorgen, Taiwan) followed by polymerase chain reaction (PCR), using the modified protocol by Godiksen *et al*. [17]. Briefly, the forward primer was 5′-AGCCTTCAGCAAGAAGCCA-3′, and the reverse primer was 5′-CAAACTTGACCTTGGA-GGAGC-3′. The PCR process was also conducted using a Thermocycler T-Gradient ThermoBlock (Biometra, Thailand). First, PCR steps were conducted at 95°C for 15 min, 35 cycles of 95°C for 30 s, 58°C for 30 s, 72°C for 1 min, and final extension at 72°C for 10 min. Then, PCR products at 242 bp on a 1.5% agarose gel electrophoresis were detected and purified using a PCR product purification kit (GEL/PCR Purification Kit, Favorgen) followed by storage at −20°C. Next, Sanger’s sequencing was performed to detect the PCR product’s nucleotide with a specific forward and reverse primer. Finally, *MYBPC3* p.A31P and p.A74T polymorphisms were detected using BioEdit 7.2 (https://bioedit.software.informer.com/) and A plasmid Editor programs (jorgensen.biology.utah.edu/wayned/ape/).

### Echocardiography

**Figure-1:**
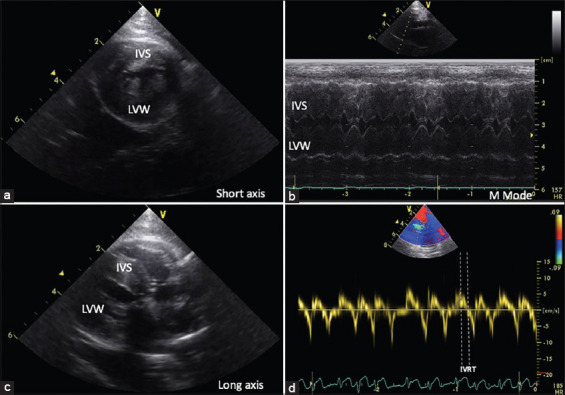
Illustration of echocardiographic recording in HCM affected Maine Coon cats. (a and c) The left ventricular wall is visualized by two-dimensional echocardiography in the short- and long-axis view. (b) Representation of HCM cat; IVS=Interventricular septum, LVW=Left ventricular wall, (d) represented IVRT=Isovolumic relaxation time, HCM=Hypertrophic cardiomyopathy.

Echocardiography (vivid 5s, GE Healthcare, USA) was routinely examined with continuous electrocardiography recording. First, brightness mode (B-mode) and motion mode (M-mode) echocardiography were used to verify the left ventricular structure on the right parasternal short-axis view at the papillary muscle level. The left ventricular hypertrophy was defined as the left ventricular wall with more than 6 mm thickness [16]. Next, the left atrium and aorta (LA/AO ratio) diameter was determined at the right parasternal short-axis view near the heart base of the AO. Then, the dynamic obstruction at the left ventricular outflow tract was examined. In addition, while the pulse wave Doppler was measured as the E/A ratio, isovolumic relaxation time (IVRT) was measured at the left parasternal, apical four-chamber view, as shown in Figure-1.

### Statistical analysis

All values were reported as mean±standard error of the mean (SEM). Clinical and echocardiographic parameters were compared between the three groups of no mutation, *MYBPC3* p.A31P mutation, and *MYBPC3* p.A74T mutation through one-way analysis of variance, followed by Tukey’s *post hoc* test using GraphPad Prism 8 (GraphPad Software, San Diego, CA, USA.). Pearson’s correlation was then used to test the correlation between age, body weight, and interventricular septum thickness at end-diastole. In addition, odds ratios for developing HCM were generated for positive *MYBPC3* p.A31P or p.A74T mutations. Moreover, the efficacy of the diagnostic test was represented by calculating sensitivity and specificity. p<0.05 was considered the minimum level of statistical significance.

## Results

**Figure-2:**
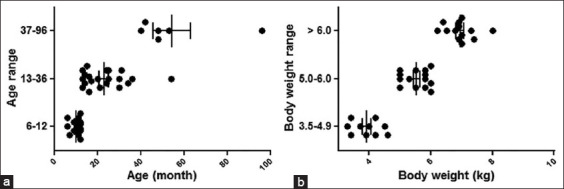
The correlation of age and body weight range of Maine Coon cats. (a) Range of age in Maine Coon cats. (b) Range of body weight in Maine Coon cats. Each dot plot represents individual cats. Pearson’s correlation test was performed.

Clinical and echocardiographic characteristics for all cats are reported in Table-1. Results showed that the average age of the cats was 24.72±3.45 months, and their average body weight was 6.13±0.38 kg, of which 23 (51.11%) were male. However, no significant difference was observed in sex or weight between the groups. Results also showed that cats with an A31P mutation were significantly older than those in the A74T mutation group (34.57 vs. 20.44 months, p=0.0025). Concomitantly, 32.26% of cats were aged 6-12 months, 45.16% were aged 13-36 months, and 22.58% were aged >36 months (Figure-2). DNA sequencing results for A31P and A74T mutations in Maine Coon cats are shown in Table-2. Our findings from genetics tests using DNA sequencing indicated that the prevalence of A31P and A74T was 16.33% and 22.45%, respectively. Moreover, the odds ratio of A31P was 0.72, and that of A74T was 0.81.

**Table 1:** Clinical and echocardiographic characteristics of Maine Coon cats (wild type; n=33, and *MYBPC3* mutation; n=12).

Clinical parameters	Wild-type cats	Mutation		p-value

		A31P	A74T	
Age in months	24.72±3.45	34.57±13.07	20.44±3.39	0.3358
Weight (kg)	6.13±0.38	5.75±2.35	5.41±0.53	0.9699
Male (number [%])	25 (51.02%)	50.00%	54.54%	-
Heart rate (bpm)	128.4±5.44**	165.17±6.74**	145.67±5.01**	0.0010
SBP (mmHg)	105.5±9.53	126.25±6.31	115.5±1.85	0.2164
Echocardiography				
LA diameter (cm)	1.19±0.08	1.31±0.54	1.38±0.18	0.9746
LA/AO ratio	1.42±0.07	1.23±0.50	1.28±0.09	0.6535
IVSd (cm)	0.63±0.03	0.56±0.23	0.56±0.08	0.6948
LVPWd (cm)	0.69±0.04	0.59±0.24	0.71±0.10	0.6327
LVIDd (cm)	1.56±0.07	1.60±0.65	1.79±0.18	0.7562
IVSs (cm)	0.74±0.03	0.65±0.27	0.66±0.07	0.7144
LVPWs (cm)	0.78±0.04	0.67±0.27	0.71±0.07	0.4946
LVIDs (cm)	0.93±0.06	1.0±0.40	1.16±0.13	0.4917
Fractional shortening (%)	40.47±1.68	37.51±1.53	35.72±2.42	0.3376
Mitral E/A ratio	0.96±0.03	0.92±0.41	0.93±0.08	0.9518
IVRT (ms)	52.09±2.48*	72.0±3.21*	62.0±7.07*	0.0110

Data are presented as mean±SEM;

* p<0.05,

** p<0.01. LA=Left atrium, AO=Aorta, IVSd=Interventricular septal at end-diastole, LVPWd=Left ventricular free proximal wall diameter at end-diastole, LVIDd=Left ventricular internal diameter at end-diastole, IVSs=Interventricular septal at end-systole, LVPWs=Left ventricular free proximal wall diameter at end-systole, LVIDs=Left ventricular internal diameter at end-systole, IVRT=Isovolumic relaxation time

**Table 2:** DNA sequencing results for A31P and A74T SNP in Maine Coon cats.

SNP	Genotype	Phenotype		n	Prevalence (%)	OR	95%CI

		Normal	Hypertrophic cardiomyopathy				
A31P	G/G	28	13	41	16.33	0.7179	0.1272-4.0507
	G/C+C/C	6	2	8			
A74T	G/G	26	12	38	22.45	0.8125	0.1826-3.6155
	G/A+A/A	8	3	11			

OR=Odds ratio, G/G=Homozygous wild type, G/C=Heterozygous mutation, C/C=Homozygous mutation

**Figure-3:**
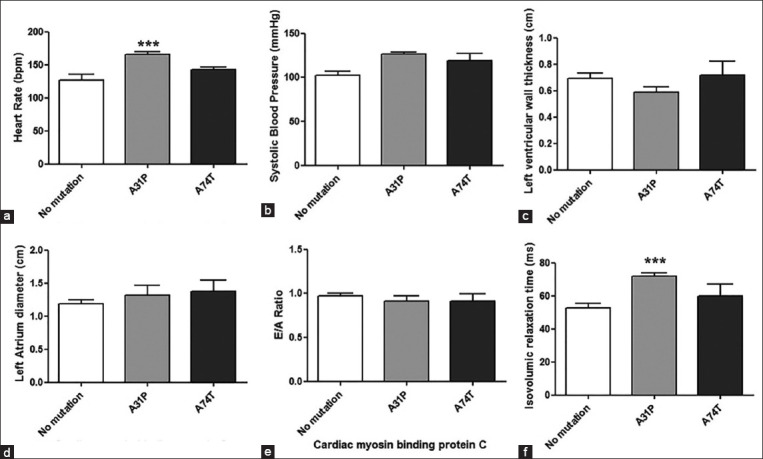
Presentation of clinical and echocardiographic parameters related hypertrophic cardiomyopathy Maine Coon cats. (a) Heart rate (bpm), (b) systolic blood pressure (mmHg), (c) left ventricular wall thickness (cm), (d) left atrial diameter (cm), (e) mitral valve E/A ratio, and (f) isovolumic relaxation time (ms) of Maine Coon cats with and without sarcomeric gene mutation. All data expressed as mean±SEM. One-way analysis of variants analyzed data with Tukey’s multiple comparison test, ***p<0.001.

**Figure-4:**
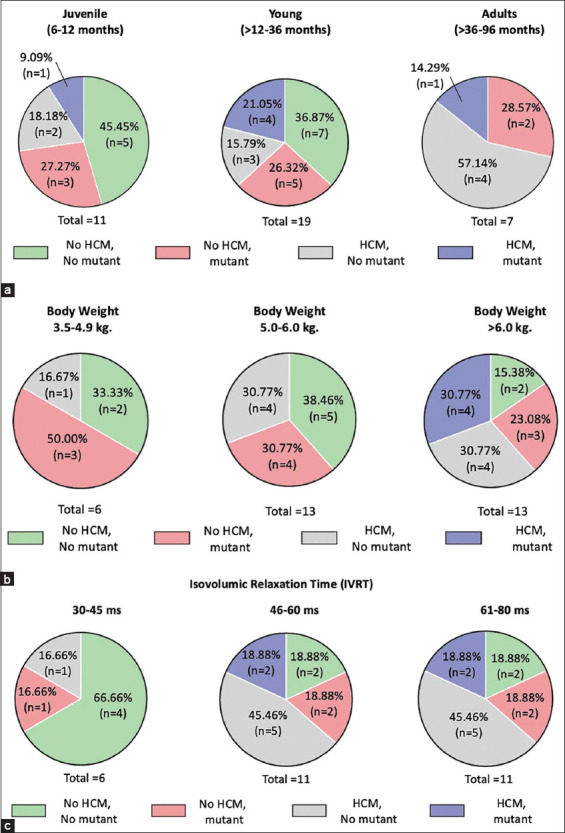
Percentage of age, body weight, and isovolumic relaxation time (IVRT) in hypertrophic cardiomyopathy Maine Coon cats. (a) Age was divided into three ranges of juvenile (6-12 months), young (>12-36 months), and adult (>36-96 months). (b) Body weight was divided into three ranges of 3.5-4.9 kg, 5.0-6.0 kg, and >6.0 kg. (c) IVRT was divided into three ranges of 30-45 ms, 46-60 ms, and 61-80 ms.

In addition, echocardiographic results are presented as the mean±SEM (Table-1). As observed, no differences existed between cats with the A31P or A74T mutations. Comparison analysis between Maine Coon cats with the A31P and A74T mutations was also performed to corroborate these results. It has been reported that cats with A31P and A74T mutations had a significantly higher heart rate and prolonged IVRT than wild-type cats. However, the results from this study did not significantly differentiate groups based on their systolic blood pressure, left ventricular wall thickness, left atrial diameter, or E/A ratio (Figure-3). Nevertheless, HCM prevalence increased with IVRT for more than 46 ms. In addition, mutation prevalence dramatically increased in cats with a higher level of IVRT having low sensitivity and specificity (Table-3). Percentages of age, body weight, and IVRT in HCM Maine Coon cats are presented in Figure-4. The prevalence of HCM was also potentially elevated with increasing age. As observed, although the occurrence of HCM considerably increased between 27.27% in the juvenile group, 36.84% in the young group, and 71.43% in the adult group (Figure-4a), the prevalence of HCM was elevated with increasing body weight (Figure-4b). In contrast, the frequency of HCM using the IVRT parameter fluctuated between the groups (Figure-4c). These results indicated that the presence of prolonged IVRT of more than 46 ms, age between 12 and 96 months, and a body weight of more than 5 kg were risk factors accounting for HCM (Figures-2 and 4).

**Table 3:** Isovolumic relaxation time parameter, the prevalence for A31P and A74T mutation, sensitivity and specificity of isovolumic relaxation time to detect HCM in Maine coon cats.

Isovolumic relaxation time (ms)	30-45 (%)	46-60 (%)	61-80 (%)
Mutation prevalence	14.29	36.36	50.00
HCM prevalence	14.29	63.63	58.33
Sensitivity	0.00	42.86	42.86
Specificity	83.33	75.00	40.00

HCM=Hypertrophic cardiomyopathy

## Discussion

This study evaluated the prevalence of *MYBPC3*, A31P, and A74T polymorphisms in Maine Coon cats and investigated risk factors accounting for HCM in cats. The previous studies have revealed that the prevalence of single-nucleotide polymorphisms in *MYBPC3* was 34-42% for A31P and 35% for A74T in Maine Coon cats worldwide [3-5]. In this study, the prevalence of these single-nucleotide polymorphisms in *MYBPC3* was 16.33% for A31P and 22.45% for A74T. However, the odds ratios were 0.72 for A31P and 0.81 for A74T. Compared with the previous studies [9,10], the prevalence of *MYBPC3*, A31P, and A74T polymorphisms and the odds ratio was lower in Maine Coon cats with HCM. Furthermore, the occurrence reported in our study was lower than these studies, which can be due to more selective breeding from Maine Coon breeders and convenient genetic testing. However, the HCM prevalence in this study was 30.63%, which was much higher than that previously reported (14-16%) [9,10].

According to our data, HCM prevalence with sarcomeric gene mutations increased with age, and an age of more than 12 months was correlated with increased left ventricular wall thickness. This finding agrees with a previous report that increasing age causes thickening of the left ventricle wall in those with an *MYBPC3*-A31P homozygous mutation [4,6]. Moreover, male cats were more expected to have sarcomeric gene mutations, which had been reported previously [8]. Furthermore, obesity is proposed as the risk factor for HCM in cats [18]. Similarly, this study observed that more than 5 kg bodyweight was related to the left ventricular wall thickness (Figure-3). In a recent publication, *MYBPC3*-R820W SNPs were associated with HCM, and body weight was strongly correlated with increased left ventricular proximal wall thickness, left ventricular dimension, and LA/AO ratio. However, no association between sarcomeric gene mutations and left ventricular wall thickness was observed in this study. Previous studies suggested that although the left atrial dilation was a significant risk factor for cardiac death in cats with HCM, the left atrial enlargement can reflect an elevated left ventricular diastolic filling pressure and diastolic dysfunction in HCM patients [20,21]. As observed, the most common factor associated with feline HCM is sudden cardiac death, similar to human HCM. Furthermore, heart functions, such as electrocardiography and echocardiography, are essential diagnostic tools to detect and evaluate many cardiovascular abnormalities. Therefore, a diagnosis would be possible if average values are established for the specific breed [22,23]. Furthermore, in this study, the LA dilation was proposed to be associated with A74T sarcomeric gene mutations (Figure-3). Hence, an *MYBPC3*-A74T mutation can reflect another cardiomyopathy phenotype, such as restrictive cardiomyopathy. Nevertheless, a larger sample size in further studies should confirm these results.

Previous evidence has suggested IVRT and the E/A ratio as important echocardiography parameters to indicate the impairment of diastolic functions in cats with HCM that have an *MYBPC3*-A31P mutation [2]. Besides, studies have reported diastolic myocardial motion as the functional echocardiographic parameter for the early detection of feline HCM [1], similar to our results. Studies have also noted that the low sensitivity of *MYBPC3*-A31P and A74T testing is a challenge for diagnosing HCM in the feline population.

## Conclusion

It can be concluded that HCM prevalence with sarcomeric gene mutations increased with age, body weight, heart rate, and IVRT. In addition, this study also demonstrated that *MYBPC3*-A31P and A74T mutations occurred at a high frequency in Maine Coon cats, associated with tachycardia and prolonged relaxation time from echocardiography. Therefore, results proposed that further investigation using diastolic myocardial motion as an early indicator of HCM in cats and screening *MYBPC3* mutations in cats is needed, especially in Maine Coon cats, as a diagnostic tool for breeding purposes.

## Authors’ Contributions

SP: Performed the echocardiography, interpreted the data and results, and manuscript writing and revision. SP and PS: Manuscript writing and figure preparation. Both authors read and approved the final manuscript.
